# Investigation of Band Alignment for Hybrid 2D-MoS_2_/3D-β-Ga_2_O_3_ Heterojunctions with Nitridation

**DOI:** 10.1186/s11671-019-3181-x

**Published:** 2019-12-02

**Authors:** Ya-Wei Huan, Ke Xu, Wen-Jun Liu, Hao Zhang, Dmitriy Anatolyevich Golosov, Chang-Tai Xia, Hong-Yu Yu, Xiao-Han Wu, Qing-Qing Sun, Shi-Jin Ding

**Affiliations:** 1 State Key Laboratory of ASIC and System, School of Microelectronics, Fudan University, Shanghai, 200433 China; 2 Key Laboratory of Micro and Nano Photonic Structures, Department of Optical Science and Engineering, Fudan University, Shanghai, 200433 China; 3 Belarusian State University of Informatics and Radioelectronics, P. Brovka street, 6, 220013 Minsk, Belarus; 4 Key Laboratory of Materials for High Power Laser, Shanghai Institute of Optics and Fine Mechanics, Chinese Academy of Sciences, Shanghai, 201800 China; 5 Department of Electrical and Electronic Engineering, Southern University of Science and Technology, Shenzhen, 518055 China

**Keywords:** Nitridation treatment, Band alignment, Few-layer MoS_2_, β-Ga_2_O_3_

## Abstract

Hybrid heterojunctions based on two-dimensional (2D) and conventional three-dimensional (3D) materials provide a promising way toward nanoelectronic devices with engineered features. In this work, we investigated the band alignment of a mixed-dimensional heterojunction composed of transferred MoS_2_ on β-Ga_2_O_3_($2-$01) with and without nitridation. The conduction and valence band offsets for unnitrided 2D-MoS_2_/3D-β-Ga_2_O_3_ heterojunction were determined to be respectively 0.43 ± 0.1 and 2.87 ± 0.1 eV. For the nitrided heterojunction, the conduction and valence band offsets were deduced to 0.68 ± 0.1 and 2.62 ± 0.1 eV, respectively. The modified band alignment could result from the dipole formed by charge transfer across the heterojunction interface. The effect of nitridation on the band alignments between group III oxides and transition metal dichalcogenides will supply feasible technical routes for designing their heterojunction-based electronic and optoelectronic devices.

## Background

Beta-gallium oxide (β-Ga_2_O_3_) has attracted considerable interests due to its superior material properties [1, 2]. With ultra-wide bandgap (4.6–4.9 eV), the theoretical breakdown electric field (*E*_*C*_) is estimated to be around 8 MV/cm [3, 4]. Combined with its high relative dielectric constant (ε) and electron mobility (μ), the Baliga’s figure of merit ($\upvarepsilon \upmu {E}_C^3$) is triple that of GaN or SiC, reducing the conduction loss significantly [1]. In addition, the availability of large bulk single crystals synthesized via melt-growth and epitaxial techniques delivers significant advantages for industrial applications [5, 6]. By far, β-Ga_2_O_3_ has been well demonstrated in a wide range of electronic applications, including light-emitting diodes, gas sensors, photodetectors, as well as field-effect transistors [7–10]. Very recently, hybrid heterojunctions, i.e., the integration of two-dimensional (2D) materials with three-dimensional (3D) materials, are of particular interest due to the complementary properties of their material systems [11].

To date, diverse 2D layered materials have been stacked on wide bandgap semiconductors to construct hybrid heterojunctions for novel applications with varying functionalities, such as MoS_2_/GaN, WSe_2_/GaN, MoS_2_/SiC, and so on [12–15]. Structurally, the MoS_2_ crystal is composed of a Mo atomic layer sandwiched between two sulfur layers, forming a two-dimensional hexagonal trilayer which is bonded to its neighboring layers by weak van der Waals forces [16, 17]. Unlike graphene with a zero bandgap, the thickness-dependent modulation of bandgaps motivated the exploration of MoS_2_ in optical and electrical devices [18, 19]. Based on the physics of MoS_2_, the density of states of few-layer MoS_2_ is three orders of magnitude higher than that of single-layer (SL) MoS_2_, leading to high drive currents in the ballistic limit. In this context, few-layer MoS_2_ may deliver significant advantages for transistor applications than SL MoS2 [18]. Thus, the integration of MoS_2_ with β-Ga_2_O_3_ is of great interest for combining respective merits of both the established 2D and 3D materials. And the optical and electrical properties for hybrid heterojunctions are inherently dominated by the interfacial energy band alignment. Consequently, it is quite desirable to have tunable band alignments for improving the performance of heterojunction based devices. In this work, we investigated the band alignment of 2D-MoS_2_/3D-β-Ga_2_O_3_ heterojunctions with and without nitridation treatment via X-ray photoelectron spectroscopy (XPS) characterizations and first principles calculations.

## Methods

**Fig. 1:**
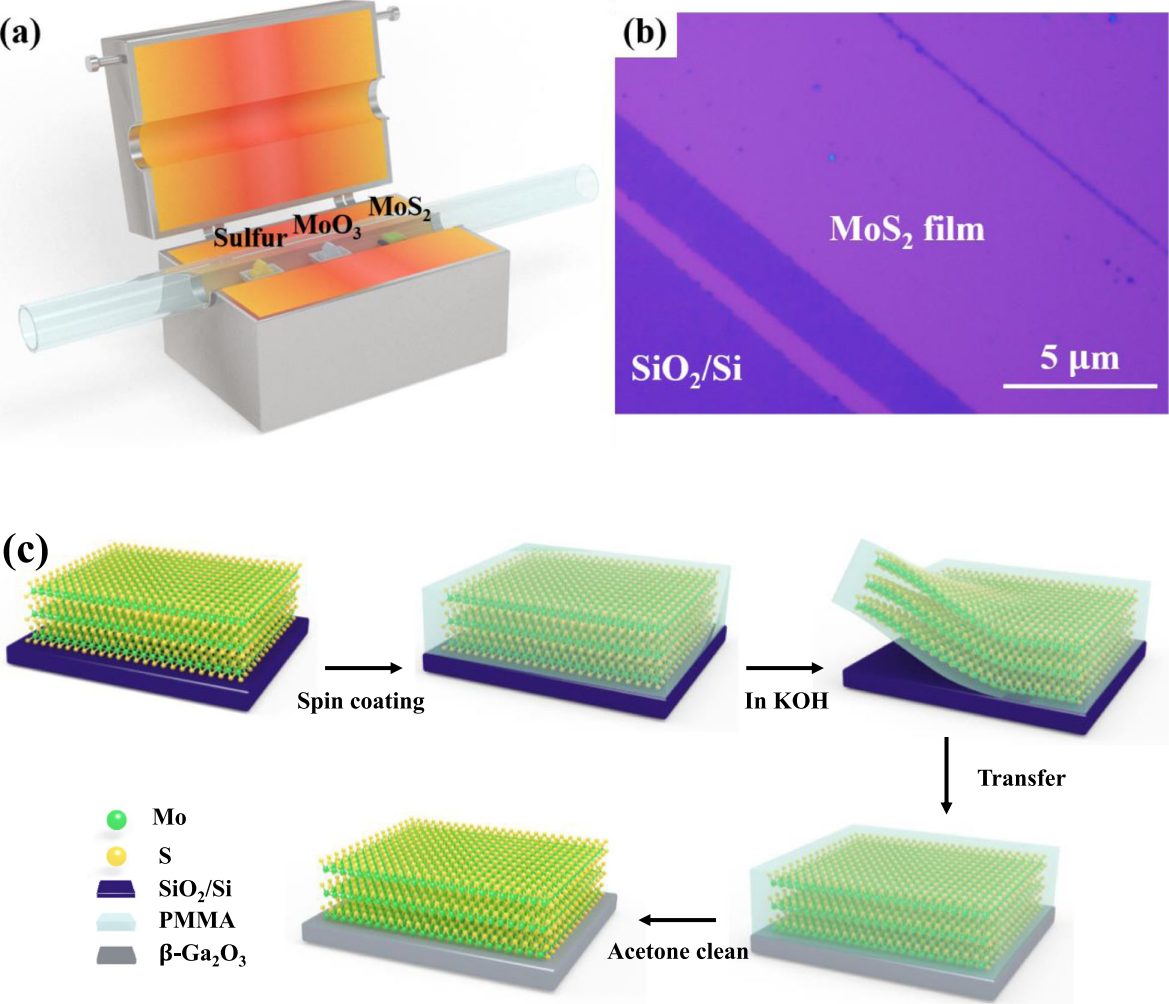
**a** Schematic illustration of the experimental set-up for CVD-growth of MoS_2_. **b** Optical image for the as-grown few-layer MoS_2_ film on SiO_2_/Si substrate. **c** Process flow of PMMA-assisted wet-transfer method for the MoS_2_/β-Ga_2_O_3_ heterojunction formation

The SiO_2_/Si substrate was ultrasonicated with acetone and visopropanol for each 10 min, respectively, followed by rinsing in deionized water and drying with N_2_. Few-layer MoS_2_ films were grown on the SiO_2_/Si substrate by chemical vapor deposition (CVD) using precursors of MoO_3_ (0.08 mg, 99%, Alfa Aesar) and S powder (1 g, 99%) [20, 21]. The MoO_3_ and S powder were placed into two separate crucibles with a SiO_2_/Si substrate in the quartz tube, as shown in Fig. 1a. During the growth process, the quartz tube was held at 800 °C for MoS_2_ film growth within 5 min. Figure 1b displays the optical microscopic image of uniform MoS_2_ film on SiO_2_/Si substrate. After the growth of MoS_2_ film, it would be transferred to β-Ga_2_O_3_ (Tamura Corporation, Japan) substrate via PMMA-assisted method, [22] as sketched in Fig. 1c. During the transfer process, PMMA was first spin-coated on as-grown MoS_2_ film as a supporting layer, and then the samples were immersed in KOH solution for etching away the SiO_2_ layer. Subsequently, the PMMA layer with MoS_2_ film would float on the solution, after which the sample would be rinsed in deionized water for 1 min to remove the residual K^+^ and further transferred onto β-Ga_2_O_3_ substrate. Lastly, the top PMMA layer would be removed away with acetone. For the nitrided MoS_2_/β-Ga_2_O_3_ heterojunction, the nitridation has been implemented on the β-Ga_2_O_3_ surface with 50s N_2_ plasma treatment at a pressure of 3 Pa prior to the MoS_2_ transfer. The RF power and N_2_ flow rate were 100 W and 80 sccm, respectively. As a result, four samples were prepared for XPS measurements: (1) uncoated β-Ga_2_O_3_ substrate (bulk β-Ga_2_O_3_), (2) few-layer MoS_2_ film on SiO_2_/Si substrate (few-layer MoS_2_), (3) transferred MoS_2_ film on β-Ga_2_O_3_ substrate, (4) transferred MoS_2_ film on nitrided β-Ga_2_O_3_ substrate.

## Results and Discussions

**Fig. 2:**
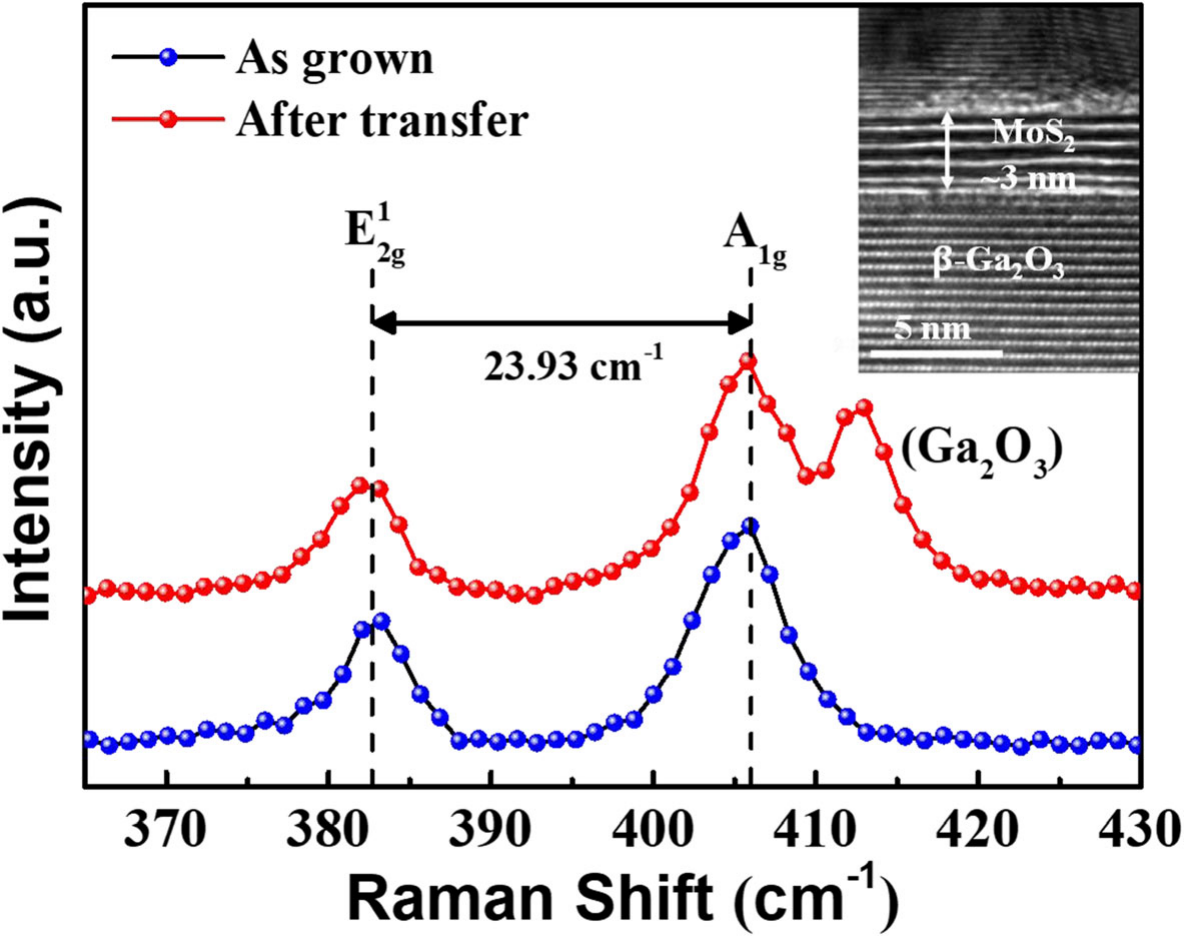
Raman spectra of as-grown MoS_2_ on SiO_2_/Si substrate and transferred MoS_2_ on β-Ga_2_O_3_ substrate, respectively. The inset shows cross-section transmission electron microscopy (TEM) image of fabricated MoS_2_/β-Ga_2_O_3_ heterojunction

**Fig. 3:**
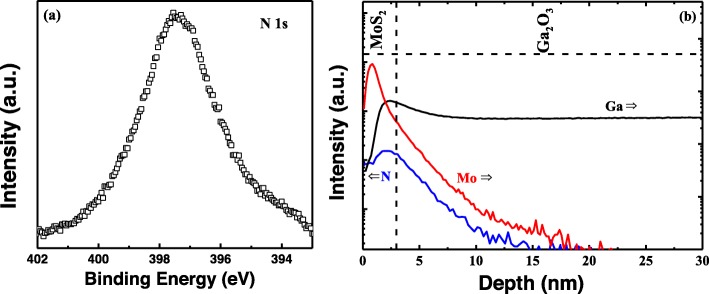
**a** N 1 s XPS spectrum of β-Ga_2_O_3_ substrate with surface nitridation. **b** SIMS depth profile of fabricated MoS_2_/β-Ga_2_O_3_ heterojunction

Raman spectroscopy was employed to investigate the quality of few-layer MoS_2_ film as well as to check relevant layer numbers. The Raman spectra of MoS_2_ film before and after transfer are presented in Fig. 2, which was characterized by RENISHAW inVia Raman spectroscopy. Two characteristic Raman modes could be observed around 381.91 cm^−1^ and 405.84 cm^−1^, corresponding to the in-plane (${E}_{2g}^1$) mode and out-of-plane (*A*_1*g*_) mode, respectively [23, 24]. Compared with as-grown MoS_2_ film, there is almost no Raman shift in ${E}_{2g}^1$ and *A*_1*g*_ modes after transfer process, indicative of undamaged MoS_2_ after transfer process. The peak at 412.99 cm^−1^ after transfer process stems from the β-Ga_2_O_3_ substrate, in consistent with previous reports [25]. The frequency difference between ${E}_{2g}^1$ and *A*_1*g*_ mode was deduced to be about 23.93 cm^−1^, designating four layers of few-layer MoS_2_ film [26]. Further, as shown in the inset of Fig. 2, the thickness of MoS_2_ film was verified to be 3 nm approximately (around four layers) by high-resolution transmission electron microscope (HRTEM), which is in good agreement with our Raman spectra. It can be seen from Fig. 3a that a high intensity peak of N 1 s was detected from the nitride β-Ga_2_O_3_ substrate, suggesting the presence of nitrogen. Figure 3b shows the SIMS profiles of MoS_2_/β-Ga_2_O_3_ heterojunction with nitridation, where the signals of main components represented by Mo, N, and Ga are plotted against depth. It is observed that the N peak is located at the MoS_2_/β-Ga_2_O_3_ interface, and the N spreading into β-Ga_2_O_3_ substrate could be contributed by the N injection into the underlying layer during plasma treatment or primary beam bombardments. The higher Ga profile in the MoS_2_ layer than β-Ga_2_O_3_ substrate probably stems from the different ion yield in the different material matrix [27]. Moreover, the tail of Mo in β-Ga_2_O_3_ could be ascribed to the diffusion or depth resolution problem, which is caused by primary beam bombardment [28].

**Fig. 4:**
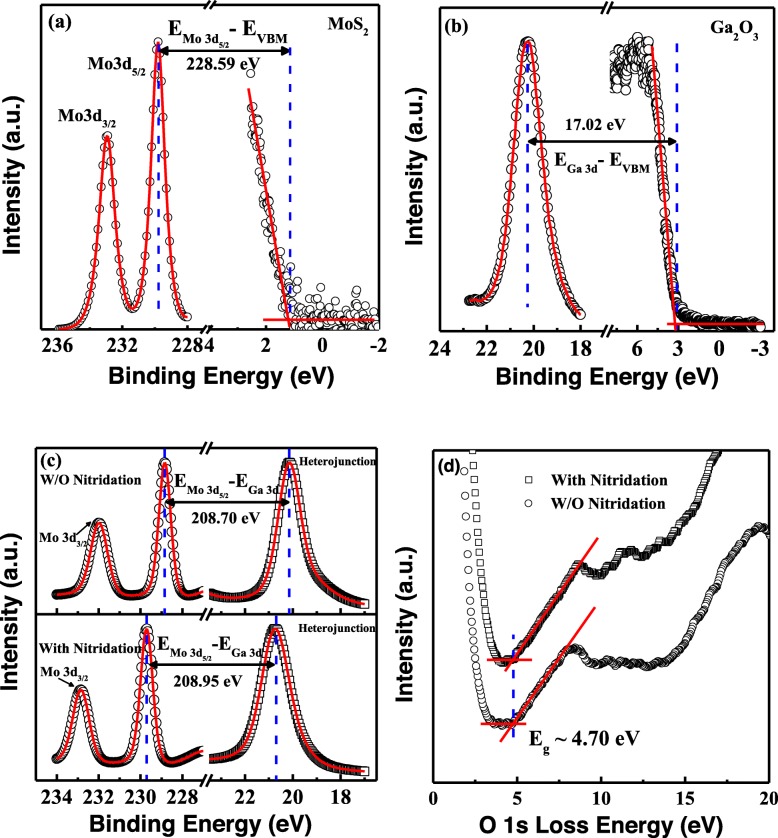
**a** XPS spectra of Mo 3d CL and valence band from few-layer MoS_2_. **b** XPS spectra of Ga 3d CL and valence band from β-Ga_2_O_3_ substrate. **c** XPS spectra of Mo 3d and Ga 3d CLs for fabricated MoS_2_/β-Ga_2_O_3_ heterojunction with/without surface nitridation. **d** XPS spectra of O 1 s CL energy loss of β-Ga_2_O_3_ substrate with/without surface nitridation

To obtain the band alignments of MoS_2_/β-Ga_2_O_3_ heterojunctions, XPS measurements with a step of 0.05 eV were carried out on VG ESCALAB 220i-XL system with a monochromatic Al Kα X-ray source (hν = 1486.6 eV). The constant pass energy was set at 20 eV. Additionally, the standard C 1 s (284.8 eV) was used for binding energy (BE) calibration [29]. To evaluate the valence band offset (VBO) at the MoS_2_/β-Ga_2_O_3_ interface, Mo 3d and Ga 3d core levels (CLs) were used for few-layer MoS_2_ and β-Ga_2_O_3_ samples, respectively. Figure 4a shows the XPS narrow scan of Mo 3d and valence band spectra from few-layer MoS_2_ [30]. The binding energy difference (BED) between CLs of Mo 3d_5/2_ and valence band maximum (VBM) for MoS_2_ was calculated to be 228.59 ± 0.1 eV. As shown in Fig. 4b, the BE of Ga 3d CL and VBM from few-layer β-Ga_2_O_3_ were deduced to be 20.25 ± 0.05 and 3.23 ± 0.05 eV, respectively. The corresponding BED was determined to 17.02 ± 0.1 eV, which is well consistent with that reported by Sun et al. [31]. Figure 4c depicts the measured XPS spectra of Mo 3d and Ga 3d CLs for MoS_2_/β-Ga_2_O_3_ heterojunctions with/without nitridation. It is noted that the Mo 3d_5/2_ CL shifted from 228.95 ± 0.05 eV for the unnitrided heterojunction toward 229.60 ± 0.05 eV for the nitrided heterojunction while Ga 3d CL shifted from 20.25 ± 0.05 to 20.65 ± 0.05 eV. Based on Kraut’ method,[32] the valence band offset (VBO, *∆E*_*V*_) of few-layer MoS_2_/β-Ga_2_O_3_ heterojunctions was calculated according to the following equation,

1 $$ \Delta  {E}_V=\left({E}_{Mo\ 3{d}_{5/2}}^{Mo{S}_2}-{E}_{VBM}^{Mo{S}_2}\right)-\left({E}_{Ga\ 3d}^{Ga_2{O}_3}-{E}_{VBM}^{Ga_2{O}_3}\right)-{\Delta  E}_{CL} $$

where ${E}_{Mo\ 3{d}_{5/2}}^{Mo{S}_2}$ and ${E}_{VBM}^{Mo{S}_2}$ are binding energies of Mo 3d_5/2_ CL and VBM from MoS_2_, ${E}_{Ga\ 3d}^{Ga_2{O}_3}$, and ${E}_{VBM}^{Ga_2{O}_3}$ are binding energies of Ga 3d CL and VBM from β-Ga_2_O_3_, ${\Delta  E}_{CL}=\Big({E}_{Mo\ 3{d}_{5/2}}^{Mo{S}_2}-{E}_{Ga\ 3d}^{Ga_2{O}_3}$) is the binding energy difference between Mo 3d_5/2_ and Ga 3d CLs for MoS_2_/β-Ga_2_O_3_ heterojunctions. Hence, the *∆E*_*V*_ of MoS_2_ on β-Ga_2_O_3_ substrate with and without N_2_ plasma treatment was calculated to be 2.62±0.1 and 2.87 ± 0.1 eV, respectively.

**Fig. 5:**
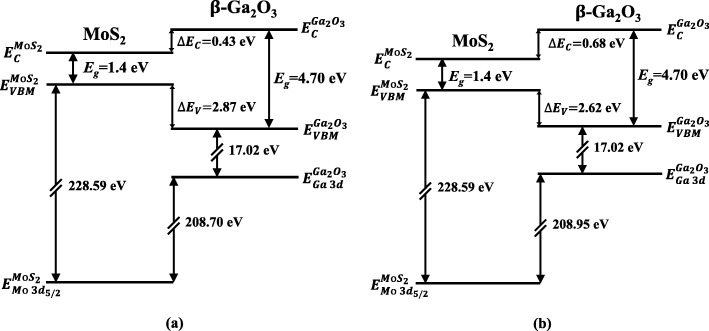
Band diagrams of MoS_2_/β-Ga_2_O_3_ heterojunction **a** without and **b** with surface nitridation

Figure 4d shows the O 1 s CL energy loss spectra of β-Ga_2_O_3_ substrates with and without nitridation. It is noted that the bandgap keeps unchanged after nitridation treatment with a value of 4.70 ± 0.1 eV. Thus, the conduction band offset can be extracted as follows,

2 $$ {\Delta  E}_C={E}_g^{Ga_2{O}_3}-{E}_g^{Mo{S}_2}-{\Delta  E}_V $$

where ${E}_g^{Ga_2{O}_3}$ and ${E}_g^{Mo{S}_2}$ are the bandgaps of β-Ga_2_O_3_ and few-layer MoS_2_, respectively. The bandgap of 1.4 ± 0.1 eV for few-layer MoS_2_ was used in this work.^34^ According to Eq. (2), the *∆E*_*C*_ between MoS_2_ and β-Ga_2_O_3_ with and without nitridation were deduced to be 0.68 ± 0.1 and 0.43 ± 0.1 eV, respectively. The calculated band diagrams for heterojunctions without/with nitridation are shown in Fig. 5(a) and 5(b), respectively.

Next, the electronic structures of nitrided and unnitrided heterojunctions were further examined through the Vienna ab initio simulation package (VASP) based on density functional theory (DFT) [33–35]. The generalized gradient approximation (GGA) of Perdew-Burke-Ernzerhof (PBE) parameterization was adopted for exchange-correlation function [36, 37]. We used the DFT-D3 dispersion corrections approach to describe the long-distance van der Waals (vdW) interactions [38–40]. The projector augmented wave (PAW) pseudopotential method was used to describe the core-valence interaction with a kinetic energy cutoff of 650 eV for plane wave expansion. We employ a 4 × 4 × 1 G-centered k-mesh for structural relaxation of the unit cell, with the smallest spacing between k-points of 0.04 Å^−1^, which is precise enough by the convergence test with respect to the number of k points. The convergence thresholds are set to 10^−4^ eV for energy differences of the system and 10^−2^ eV Å^−1^ for Hellman-Feynman force. In order to eliminate artificial interactions between two adjacent atomic layers, the thickness of the vacuum layer is set to ~ 15 Å. The eigenvalues of the heterojunctions are further verified by the Heyd-Scuseria-Ernzerhof (HSE06) hybrid functional calculations, which improve the precision of eigenvalues via reducing the localization and delocalization errors of PBE and Hartree-Fock (HF) functionals [41]. The mixing ratio is 25% for the short-range HF exchange. The screening parameter is 0.2 Å^−1^.

**Fig. 6:**
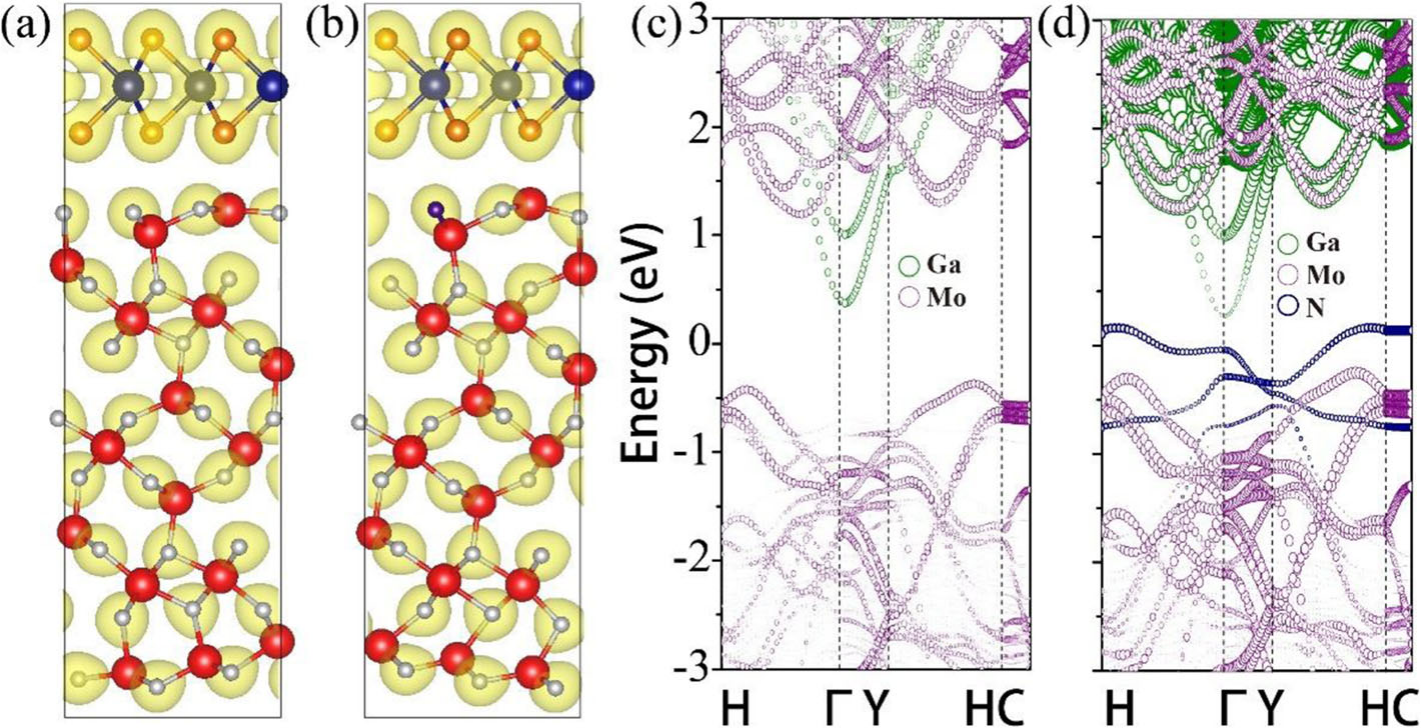
Atomic structure and charge-density distributions of β-Ga_2_O_3_-MoS_2_ stacked heterostructures **a** without and **b** with nitrogen dopants in a 4 × 4 × 1 supercell from a side view. Ga (O) atoms are in red (gray) and Mo (S) atoms in blue (orange). Band structures of MoS_2_/β-Ga_2_O_3_ heterostructures **c** without and **d** with nitrogen dopants

The MoS_2_/β-Ga_2_O_3_ heterojunctions were constructed as shown in Fig. 6a. The universal binding energy relation (UBER) method, which provides a simple universal form for the relationship between binding energy and atomic separation, [42] was applied to determine the energetically stable structure before electronic structure calculation. Various interlayer distances were considered and the surface adhesion energy *W*_*ad*_ for the heterojunctions are shown below,

$$ {W}_{ad}=\frac{E_{Ga_2{O}_3}+{E}_{Mo{S}_2}-{\mathrm{E}}_{Ga_2{O}_3/ Mo{S}_2}}{A} $$

where A is the interface area, ${E}_{Ga_2{O}_3}$, ${E}_{Mo{S}_2}$, and ${E}_{Ga_2{O}_3/ Mo{S}_2}$ are the total energies of β-Ga_2_O_3_, monolayer MoS_2_ and the MoS_2_/β-Ga_2_O_3_ heterojunction, respectively. Once the *W*_*ad*_ reaches a maximum, the optimal interlayer distance will be obtained. After structure optimizations, a nitrogen atom is substitutionally doped in the original MoS_2_/β-Ga_2_O_3_ heterojunction, as shown in Fig. 6b. The concentration of nitrogen in DFT calculation is around 4.17%, which is close to that (3.61%) in experiments. The electronic structures for both nitrided and unnitrided MoS_2_/β-Ga_2_O_3_ heterojunctions were calculated as illustrated in Fig. 6c and d. It was seen that mid-gap states were introduced, which may enhance the charge transfer across the MoS_2_/β-Ga_2_O_3_ interface, and the resulting interface dipole contributed to the measured binding energy shift. Furthermore, the calculated conduction band offsets *∆E*_*C*_ ($\Delta  {E}_C={E}_{CB}^{Mo{S}_2}-{E}_{CB}^{Ga_2{O}_3}$) for undoped- and doped-β-Ga_2_O_3_/MoS_2_ heterojunctions are 0.82 and 1.0 eV respectively, showing the same trend with the experimental results. We have also calculated the eigenvalues of ${E}_{CB}^{Mo{S}_2}$ and ${E}_{CB}^{Ga_2{O}_3}$ using the HSE06 method to further confirm the above conclusion, and find that the corrected *∆E*_*C*_ are 0.87 and 1.08 eV for undoped- and doped-β-Ga_2_O_3_/MoS_2_ heterojunctions respectively.

## Conclusions

In conclusion, respective MoS_2_ film has been transferred onto unnitrided and nitride β-Ga_2_O_3_ for constructing MoS_2_/β-Ga_2_O_3_ heterojunctions. Raman spectroscopy was used to investigate the quality of transferred MoS_2_ film, and SIMS study was performed to probe the elemental depth profiles of the MoS_2_/β-Ga_2_O_3_ heterojunction with nitridation. The VBOs were determined to be 2.62 ± 0.1 eV for nitrided MoS_2_/β-Ga_2_O_3_ heterojunction and 2.87 ± 0.1 eV for unnitrided heterojunction by XPS, respectively. The resultant CBOs were deduced to be 0.68 ± 0.1 and 0.43 ± 0.1 eV, which was in the same trends with the DFT calculations. These findings demonstrated that the band offsets can be modified via surface nitridation process. This study offers glorious perspectives on the implementation of designed electronic devices based on 2D/3D vertical heterojunctions.

## Data Availability

The datasets supporting the conclusions of this manuscript are included within the manuscript.

## Abbreviations

β-Ga_2_O_3_: Beta-gallium oxide
SL: Single-layer
MoS_2_: Molybdenum disulfide
XPS: X-ray photoelectron spectroscopy
CBO: Conduction band offset
VBO: Valence band offset
CVD: Chemical vapor deposition
PMMA: Poly(methyl methacrylate)
HRTEM: High-resolution transmission electron microscope
SIMS: Secondary ion mass spectrometry
BE: Binding energy
BED: Binding energy difference
CL: Core level
VBM: Valence band maximum
VASP: Vienna ab initio simulation package
DFT: Density functional theory
GGA: Generalized gradient approximation
PBE: Perdew-Burke-Ernzerhof
PAW: Projector augmented wave
UBER: Universal binding energy relation
